# Experiences of COVID‐19 pandemic‐related stress among sexual and gender minority emerging adult migrants in the United States

**DOI:** 10.1002/smi.3198

**Published:** 2022-09-13

**Authors:** Edward J. Alessi, Shannon P. Cheung, Vincent Sarna, Michael P. Dentato, Andrew Eaton, Shelley L. Craig

**Affiliations:** ^1^ Rutgers The State University of New Jersey New Brunswick New Jersey USA; ^2^ Loyola University Chicago Chicago Illinois USA; ^3^ University of Regina Regina Saskatchewan Canada; ^4^ University of Toronto Toronto Ontario Canada

**Keywords:** COVID‐19, emerging adults, migration, sexual and gender minority, strength and resilience, stress

## Abstract

There is a dearth of research that examines COVID‐19‐related stress among multiply marginalised individuals who are in the developmental phase of emerging adulthood. This qualitative study investigated how the intersection of emerging adulthood, sexual and gender minority (SGM) identity, and migrant status were reflected in the experiences of SGM individuals (*n* = 37; ages 20–25 years old) who migrated to various parts of the United States in the last 5 years. Data were collected online using semi‐structured interviews. Thematic analysis revealed that participants' developmental processes (e.g., identity exploration, building financial independence) were shaped by pandemic‐related stressors, especially unemployment and financial instability. Participants who were able to maintain employment did so but at the risk of their health and safety. Findings also showed that participants experienced feelings of anxiety and depression due to social isolation, but online communication played an important role in combatting loneliness. Findings highlight the potential for trauma‐informed and intersectional approaches to practice with SGM emerging adult migrants and expanded health services and temporary entitlement programs to mitigate the pandemic's effects on this population's psychosocial and financial well‐being.

The United States ranks number one among destination countries for international migration (United Nations Population Division, 2020). Decisions to migrate are frequently motivated by a combination of factors (Czaika et al., 2021), including the freedom to express one's sexual orientation or gender identity in environments that are more accepting of sexual and gender minority (SGM) individuals (Carrillo, 2017; Hoffman & Velasco, 2021). The SGM migrant population in the United States totals 1.25 million, accounting for 3% of all migrants and 6.25% of all SGM adults in the country (Goldberg & Conron, 2021; Human Rights Campaign Foundation, 2021).

While many SGM migrants leave their countries of origin searching for safety and protection, they often continue to face interpersonal violence and social‐structural inequalities in destination countries, including the United States (Alessi et al., 2021; Golembe et al., 2021). These vulnerabilities may be compounded by stressors common to various developmental phases. For instance, it has been estimated that 42% of the SGM migrant population in the United States are in their late teens to late twenties (Goldberg & Conron, 2021). For those 18–25 years old, this period is typically referred to as emerging adulthood (Arnett, 2000). Research on SGM emerging adult migrants has been scarce, especially during the COVID‐19 pandemic, which has increased stress for marginalised groups (Kantameni, 2020). The pandemic‐related research that is available tends to examine SGM emerging adults and migrants discretely. SGM emerging adults have experienced increased isolation during the COVID‐19 pandemic due to the need for social distancing (Fish et al., 2020; Scroggs et al., 2021). Some have also had to return to homes with unsupportive family members (Mitchell et al., 2022). Migrants have also encountered severe stressors during the pandemic, including housing insecurity, unemployment, and difficulty accessing healthcare when ill (Mengesha et al., 2022). Undocumented migrants have been seriously affected, as most were ineligible for government benefits to help sustain them through extended periods of unemployment (Clark et al., 2020). Thus, the current study seeks to fill a knowledge gap by understanding the intersectional experiences of pandemic‐related stress among SGM emerging adult migrants. Results may guide service provision, programing, and policy to support SGM emerging adult migrants during public health crises.

## 1 SEXUAL AND GENDER MINORITY EMERGING ADULTS

The paradigm of life course scholarship has gradually shifted to acknowledge a new pattern of development in industrialised societies (Arnett, 2000; Salvatore, 2018), since, instead of settling into long‐term adult roles immediately, many individuals spend their late teens to mid‐twenties amassing new experiences (Arnett, 2000; Salvatore, 2018). Emerging adulthood presents opportunities to explore identity and build independence, but its transitions can be destabilising, partially explaining why this stage confers vulnerability to psychopathology (Hunt & Eisenberg, 2010). Emerging adulthood has been an evolving concept, partly due to social and economic forces making it increasing difficult for young adults who must financially support themselves, without the assistance of family, to engage in exploration that is characteristic of this phase (Hendry & Kloep, 2010; Thomas & Azmitia, 2014). In fact, evidence suggests that emerging adults experience higher rates of poverty than other age groups (Hawkins, 2019).

The study of emerging adults as a monolith can obfuscate disparities faced by subgroups during this developmental stage. Specifically, SGM emerging adults are challenged by minority stress (Brooks, 1981; Hendricks & Testa, 2012; Meyer, 2003) due to exposure to the heterosexist and cissexist contours of everyday life (Wagaman et al., 2014). Minority stress theory (Meyer, 2003) postulates that health disparities between cisgender/heterosexual individuals and SGM individuals can be explained by the unique social stressors (e.g., discrimination and prejudice, expectations of stigma, internalising stigma, identity concealment) that the latter experience as a result of their minority status (Pachankis, 2007; Reisner et al., 2016). Minority stress has been linked with negative outcomes, like depression, substance use, suicidal ideation, and unwanted sexual experiences (Hunter et al., 2021; Mongelli et al., 2019; Murchison et al., 2017).

While SGM emerging adults are at risk for these negative outcomes, they also exhibit remarkable strength and resilience in the face of adversity (Russell, 2005). For example, SGM emerging adults have leveraged information and communication technologies (ICTs) to combat the effects of minority stress, using them to access information, find community support, and develop a sense of acceptance (McInroy et al., 2019). However, ICT use has also been associated with negative outcomes among SGM youth, including higher rates of victimisation and psychological distress (McConnell et al., 2017).

## 2 INTERSECTIONALITY

While SGM emerging adults share similarities when it comes to their diverse sexual orientations and gender identities, their experiences are also shaped by systems and structures relating to race/ethnicity, class, ability, and immigration status (Moradi & Grzanka, 2017). Emerging adults with intersecting identities (e.g., being a Black gay migrant) experience stigma and discrimination that is compounded by their minoritised and racialised identities. Thus, intersectionality (Crenshaw, 1989) allows for examination of how the interplay of various forms of identity reflect interlocking systems of oppression for multiply marginalised individuals. Grounded in and informed by the work of Black feminists and women‐of‐colour activists (Collins & Bilge, 2016), intersectionality underscores how distinct forms of oppression, which are deeply embedded in societal structures, shape the individual experiences of those holding more than one marginalised identity (Moradi & Grzanka, 2017).

Research on SGM migrants has used intersectionality to explain how stigma and discrimination related to their multiply minoritised identities can make it difficult to meet basic needs and access support from diaspora communities, who can be homophobic and transphobic, and SGM and mainstream communities, who can be racist, xenophobic, as well as homophobic and transphobic (Alessi et al., 2020; Lee & Brotman, 2013).

## 3 COVID‐19 AND ITS IMPACT ON MARGINALISED POPULATIONS

Intersectionality can appropriately contextualise the stressful experiences of SGM emerging adult migrants during the COVID‐19 pandemic. The United States currently has the highest number of COVID‐19 cases and related deaths in the world (Johns Hopkins Coronavirus Resource Center, 2022), and the pandemic has worsened inequalities among marginalised groups that were already disproportionately affected by systemic oppression (Kantameni, 2020). For instance, there is evidence that the pandemic exacerbated food insecurity (Russomanno & Tree, 2020) and financial hardship (Wilson et al., 2020) for SGM individuals. Additionally, SGM adults were at high risk for unemployment, with one in three employed in industries (e.g., restaurants, transportation) vulnerable to closure (Gonzales & de Mola, 2021). Higher rates of psychopathology have also been reported among SGM people during the pandemic (Kamal et al., 2021; Salerno et al., 2020). Closures of schools and recreational spaces increased time spent in abusive homes and decreased access to identity‐based supports, which may have been contributing factors (Gato et al., 2021; Sachdeva et al., 2021). COVID‐19‐related barriers to health services also precluded the wellness of SGM individuals with intersectional identities; for example, disruption to HIV and behavioural health services may have compounded existing factors that contribute to HIV risk for Latinx sexual minority men (Harkness et al., 2022a). However, the strengths of SGM individuals throughout the pandemic must be recognised. Studies have shown that SGM youth used online supports during COVID‐19 to maintain social connections, helping them cope with adversity (Fish et al., 2020; Gonzalez et al., 2021).

The pandemic has also exacerbated social‐structural stressors for migrants, many of whom, like SGM adults, worked in COVID‐19‐sensitive industries and lost their jobs (Borjas & Cassidy, 2020). In leisure and hospitality, the April 2020 unemployment rate (39.3%) was the highest on record for the industry (U.S. Bureau of Labor Statistics, 2022). Existing policies also prevent migrants, especially undocumented migrants, from accessing healthcare or economic relief, leaving many without any formal social safety net (Mengesha et al., 2022). Meanwhile, working during the pandemic came with fears about possible exposure to COVID‐19 (Sonmez et al., 2020). However, as with SGM people, emerging evidence has shown that ICTs help migrants maintain connections with family and friends and manage pandemic‐related challenges (International Office of Migration [IOM] Diversity, Inclusion, Social Cohesion Initiative, 2020).

The convergence of vulnerabilities associated with the intersection of migration status and SGM identity is likely to have created unique stressors for SGM migrants during the pandemic. Harkness et al. (2021) found that Latino sexual minority men who were recent immigrants were more likely to be diagnosed with COVID‐19 and to experience financial loss than their U.S.‐born counterparts. The study by Harkness and colleagues serves as a primer for understanding the challenges of being at the intersection of two communities which were disproportionately affected by the pandemic.

Building on previous research, the current study is guided by three research questions: (a) How did the COVID‐19 pandemic shape the day‐to‐day experiences of SGM emerging adult migrants in the United States?; (b) How do SGM emerging adult migrants describe and understand the psychosocial distress they experienced?; and (c) What role did ICTs play for these individuals during the pandemic?

## 4 METHOD

This qualitative study was part of a larger study that explored how ICTs facilitated resettlement and integration for this population (Alessi et al., 2022). Results of the larger study illuminated the role of ICTs across participants' migration trajectory. In this article, *SGM migrant* refers to SGM immigrants, refugees, or asylum seekers. *Immigrants* are those who move to a foreign country, permanently or for an extended period, including international students (IOM, 2022). Despite having valid documentation, international students experience ‘extended precarity,’ as they must assess how to live and work in the destination country after their student visa expires (Chacko & Price, 2021). This precarity can be compounded for some SGM international students because returning to their country of origin means needing to once again conceal their identities to protect themselves against violence and abuse (Alessi et al., 2017). *Refugees* and *asylum seekers* are those who flee from their country of origin to escape persecution, but refugees are vetted prior to arrival in a host country, while asylum seekers are vetted afterwards (United Nations High Commission for Refugees, 2021a, 2021b). There certainly are differences among these groups, but SGM migrants is used as they share similar vulnerabilities across the migration trajectory (Alessi et al., 2021; Lee, 2019).

### 4.1 Participants and procedure

The sample comprised 37 SGM individuals between the ages of 20–25 (*M* = 23.27).

Table 1 displays the sample's demographic information. We protected participants' privacy and thus did not ask which U.S. state they lived in, but, in some interviews, participants reported living in various geographic areas across the continental United States.

**TABLE 1:** Demographic information for sexual and gender minority emerging adults, ages 20–25

Participant	Immigration status	Gender identity	Sexual orientation	Employment
1	U.S. citizen	Man	Gay	Yes
2	Permanent resident^a^	Man	Gay	Yes
3	Asylum seeker	Man	Gay	Yes
4	Student visa	Genderqueer/non‐binary	Queer/pansexual	No
5	Student visa	Trans woman	Bisexual	No
6	Permanent resident	Man	Gay	No
7	Permanent resident	Man	Gay	Yes
8	Permanent resident	Man	Gay	Yes
9	Student visa	Man	Gay	Yes
10	Permanent resident	Man	Gay	Yes
11	Undocumented	Woman	Gay	Yes
12	U.S. citizen	Woman	Bisexual	No
13	Permanent resident	Trans man	Bisexual	Yes
14	Student visa	Man	Gay	Yes
15	Permanent resident	Man	Bisexual	Yes
16	Asylum seeker	Man	Gay	Yes
17	Permanent resident	Trans man	Gay	Yes
18	Undocumented	Trans woman	Gay	No
19	Undocumented	Genderqueer/non‐binary	Queer	No
20	Student visa	Woman	Lesbian	No
21	Permanent resident	Woman	Bisexual	Yes
22	Declined	Man	Gay	Yes
23	Permanent resident	Man	Bisexual	No
24	Permanent resident	Woman	Bisexual	Yes
25	Asylum seeker	Man	Gay	Yes
26	Student visa	Trans woman	Bisexual	Yes
27	Permanent resident	Trans man	Gay	Yes
28	Permanent resident	Trans man	Gay	Yes
29	Asylum seeker	Genderqueer/non‐binary	Gay	Yes
30	Asylum seeker	Man	Gay	No
31	Declined	Man	Bisexual	Yes
32	Permanent resident	Man	Gay	Yes
33	Undocumented	Trans woman	Bisexual	Yes
34	Declined	Man	Gay	No
35	Permanent resident	Trans man	Gay	Yes
36	Permanent resident	Trans man	Gay	Yes
37	Permanent resident	Woman	Lesbian	Yes

*Note*: Participants identified by number to protect their identities. Some declined to report immigration status.

^a^ Being a permanent resident is also referred to as having a Green Card.

We recruited participants nationally using purposive sampling. A study announcement was emailed to community and social service agencies that provide socio‐emotional and practical support to LGBTQ+ individuals or migrants in the United States. This announcement was also posted on social media (Facebook and Instagram) with a series of keywords, or hashtags (e.g., #lgbtq, #refugeeswelcome) to increase visibility. We also paid to advertise the study on Facebook for 1 week and allowed participants to refer others for participation. Facebook advertising has been used for study recruitment (Akers & Gordon, 2018) and is useful for reaching specific populations through options to target audiences based on various characteristics, including age, geography, and interests. Inclusion criteria for this study were: (a) be 18–25 years old; (b) identify as a SGM person; (c) have migrated to the United States within the past 5 years; and (d) feel comfortable speaking conversational English. A screening questionnaire to ensure eligibility was developed as part of the study protocol. Participants who did not meet inclusion criteria or reported acute psychological distress were considered ineligible. No individuals reported psychological distress during screening, but a procedure was created to refer individuals to resources as needed (McInroy, 2016). Those deemed eligible for study participation were asked to provide a pseudonym to maintain anonymity throughout the study process. All individuals were screened by the second author via telephone before each interview; 38 of 40 individuals who were screened ultimately met eligibility criteria. Once interviews were scheduled, participants received a confirmation email with a copy of the informed consent document and were instructed to review it. This allowed participants ample time to familiarise themselves with the study and to ask any questions about participation (Newman et al., 2021). Interviewers reviewed the consent form with participants, allowed time to address questions or concerns, and obtained consent before data collection. The institutional review board of the first author's university approved all study procedures.

### 4.2 Data collection

Data collection consisted of two stages: first, a short demographic questionnaire (age, country of origin, gender identity, sexual orientation, education level, immigration status, social media platforms used), followed by semi‐structured interviews conducted via Zoom between October 2020 and March 2021. The use of Zoom as a viable method for conducting qualitative interviews is well documented (Archibald et al., 2019), and its use was vital for a national study.

Interviewers had their webcams on during the interviews, and while participants were invited to do the same, due to privacy concerns, low Internet bandwidth, or limited webcam functionality, 27 kept their webcams off. Eight participants had their webcams on, and three turned their webcams off in the middle of their interviews due to connectivity issues. One of those three interviews was not completed due to ongoing connectivity issues; thus, data from this participant was not analysed.

Interview questions included: (a) tell me about some of the reasons you came to the United States; (b) describe what it has been like to be a SGM migrant in the United States; (c) when you arrived, tell me about any support or assistance you received; (d) tell me what living through COVID‐19 has been like for you; (e) tell me about your use of ICTs from the time you arrived until now; and (f) tell me about your life now. All questions included probes to ensure that interviewers gathered data that pertained to unique participant experiences as SGM emerging adult migrants. Probes asked participants to talk more about how COVID‐19 impacted their mental health, finances, housing situation, and access to healthcare and social supports. Three doctoral research assistants who identify as SGM with interest in studying stress, health, and identity conducted the interviews. All interviewers had been trained in qualitative methods. They either conducted qualitative research in the past, took a qualitative research course, or engaged in ongoing meetings with the lead investigator (i.e., first author). Interviews lasted between 18 and 100 min (*M* = 51.1; SD = 22.4). Saturation was reached by the 25th interview, meaning that at that point, new interviews stopped providing little or no new information (Guest et al., 2020). Because 13 participants had already been recruited before saturation, we still interviewed them. As a token of appreciation, participants received a $40 Amazon gift card after completing the interview. Interviews were audio‐recorded and professionally transcribed.

### 4.3 Data analysis

We analysed the data using Braun and Clarke's (2006) principles of thematic analysis. Thematic analysis is a flexible method of identifying and reporting patterns in qualitative data (Braun & Clarke, 2006). In this study, we used it as a realist and constructionist method, reporting on meanings and realities attached to participant experiences and the ways these meanings and realities are shaped by social forces (Braun & Clarke, 2006). Analysis began with the second author (SPC) familiarising themself with the data, including previous codes that were developed from the larger study (Alessi et al., 2022). In the current study, codes were generated inductively (i.e., new codes were added to the codebook) and deductively (i.e., codes that were already identified, but not used as part of the larger study, were further explored to discern relevance to the current study's research questions). Examples of early codes include: COVID‐19 and anxiety, uncertainty, loneliness, financial instability, employment concerns, and risks to health. Once coding was complete, the first and second authors (EA and SPC) engaged in peer debriefings to generate preliminary themes. This included returning to the dataset to confirm that themes answered the research questions and conveyed a cohesive narrative. Theme names were created and refined to ensure they captured participant experiences and the meanings attached to these experiences. Themes were not finalised until both the first and second authors reached mutual agreement.

During analysis, we (EA and SPC) acknowledged our own assumptions to ensure that participant experiences were not diluted. We assumed that participants would experience challenges and subsequent distress as a result of the COVID‐19 pandemic and that the impacts would be magnified by insecure immigration status or lack of support. After identifying our key assumptions, we discussed how our positionalities (that is, as LGBTQ+ individuals with secure immigration status) might shape the development of themes. We challenged one another to be reflexive, so our standpoints did not overshadow the analysis (Barusch et al., 2011). To ensure methodological rigour, we used negative case analysis to identify participants whose experiences were not consistent with the majority. We also engaged in member checking, emailing all participants a draft of the themes and asking whether the themes captured their experiences. Seven (18.9%) responded and confirmed findings aligned with their contributions. Further, we used an audit trail to document all processes and procedures.

## 5 RESULTS

Participants reported they migrated to the United States for a combination of reasons (e.g., education, employment), with the majority stating that a primary factor was the need to live openly as person with a diverse sexual orientation or gender identity. Many hoped migrating would provide them with opportunities to live free of stigma from family and community members. The COVID‐19 pandemic shaped participant experiences as SGM emerging adult migrants in the United States. Their stress increased considerably as they struggled with financial instability, risks to their health and well‐being, immigration concerns, and feelings of isolation. We identified four themes (summarised in Table 2) to demonstrate the intersectional forms of stress that participants faced during the pandemic and the ways they manifested strength. Participant quotes are used to illustrate themes; pseudonyms were chosen by participants.^1^

**TABLE 2:** Summary of themes identifying sexual and gender minority emerging adult migrants experiences of stress during the COVID‐19 pandemic

Themes	Evidence for themes
‘It's very bad, very bad feeling:’ Dealing with anxiety and uncertainty related to COVID‐19 jobloss and insecurity	Descriptive Participants migrated for a combination of reasons (education, employment), but the need to be open about their sexual orientation or gender identity was a driving factor. Many hoped migrating would provide them with opportunities to live without facing negative reactions from family and community members. Living freely in United States meant needing to have the financial means to live on their own, and before the pandemic many participants supported themselves. However, the onset of the pandemic brought about work unpredictability and job loss, which shaped their emotional state and outlook. Participants reported feelings of anxiety and uncertainty. Some were not eligible for government benefits because of their immigration status. Others did not know whether they were eligible for benefits or how to access them.
	Exemplar quotes I'm not in my country. I'm not near my parents … my siblings … [or] my relatives. I have no job. I don't have my family here. I don't have anything that could enable [me] to get this food to eat. I don't have money to pay for my rent. It was really hard for me. (Abby, 21‐year‐old gay man). It's not a very good feeling. Losing a job, losing some way you can get some funds and buy clothes and buy shoes, can buy electronics for your own. So the job disappears. It's very bad, very bad feeling. (Tim, 24‐year‐old gay man).
‘When a job calls, what you have to do is answer:’ Navigating the risks of in‐person work during COVID‐19	Descriptive Participants had no option but to continue working outside of the home at the onset of the pandemic, as they could not rely on their parents or others for financial support. Not having much choice left participants uncomfortable about the risks they had to take to survive during a pandemic that the world did not know much about at the time, including how contagious or deadly it could be. Participants took precautions to protect themselves (e.g., social distancing, wearing masks), but the people they worked with or for did not always follow health and safety protocols, leaving participants in danger of contracting COVID‐19.
	Exemplar quotes Some clients don't want to come [in] and get the [hair styling] services done. They want you to go door‐to‐door. And so that is what we have been doing, because [the clients] feel that salons and parlours are no longer as safe as they used to be … sometimes we have to do it because it is the way that you earn a living. (Lann, a 25‐year‐old bisexual woman). I cannot stay at home … At the same time, we have this virus. It has been 1 year. I can say [it is] terrifying. But when a job calls, what you have to do is answer … (Lotus, a 23‐year‐old gay man).
‘It's just a very stressful time and there is no solution:’ Contending with loneliness, fear, and anxiety during COVID‐19	Descriptive The pandemic's consequences were far‐reaching, going beyond the negative feelings that were brought on by work and financial strain. COVID‐19 quarantine left participants isolated; many had already experienced isolation in their countries of origin because of their sexual orientation or gender identity. Participants reported intense loneliness, fear, and anxiety. This was compounded for transgender and non‐binary participants, who were dealing with gender dysphoria, preexisting mental health issues, and insecure immigration status.
	Exemplar quotes So it's very stressful. But it's also very stressful to stay in the same place. It's just a very stressful time and there is no solution. Everything is stressful and there is nothing that you can do that isn't stressful. (Luke, 22‐year‐old bisexual man). I think a lot of things hit on top of each other. Like my anxiety just got so much worse… (Juno, 23‐year‐old queer non‐binary individual). … What if I do end up in the hospital? [It] Is a really terrifying thing, because of being hospitalised for mental illness when I was here in 2016. So … dealing with that all over again. And … I have a lot of dysphoria with regards to my body in general. So having people constantly poking and prodding at me … actually scares the living crap out of me. (Archie, a 25‐year‐old non‐binary pansexual individual). I've been, like scared, even to seek … healthcare services, everything. I'm scared like they will discover and maybe deport me. So mostly I've been laying low. (John, a 21‐year‐old transgender woman).
‘We would talk about how life is going…and motivate each other about daily life:’ Using online communication to manage social isolation during COVID‐19	Descriptive Despite the challenges participants faced during the pandemic, they found a way to be resourceful by using ICTs. This highlighted participants' strength during the pandemic. ICTs (e.g.,WhatsApp, Facebook, Zoom) helped participants stay connected to other people during quarantine, which helped combat loneliness. ICTs (e.g., dating apps) could also buffer against anxiety and depression by helping participants make new connections. Participants reported a drawback to using ICTs during the pandemic; constantly reading about and hearing bad news heightened the anxiety of some.
	Exemplar quotes It was very hard during the COVID to meet someone, even travelling because of the lockdown. It has been very difficult because you have no private vehicle. So I started using … the dating apps … at least for me to socialise and have friends… (Alan, a 24‐year‐old gay man). We are able to not just support each other mentally, we kind of support each other financially sometimes. So one of us has a challenge…then we come together and collect and look for a way [to help] … we deal with collective issues that then regard each of us (Slayer, 24‐year‐old bisexual woman). I felt like it was taking a toll on me so I kind of stopped looking at the news. It's kind of selfish, but I had to just shut it down for a little bit … (Ron, a 25‐year‐old gay man).

### 5.1 Theme 1: ‘It's very bad, very bad feeling:’ Dealing with anxiety and uncertainty related to COVID‐19 job loss and insecurity

For most participants, financial autonomy was required in order to live freely in United States. and before the pandemic they maintained this autonomy through steady employment in the service industry (hairstyling, cleaning, and providing transport). However, the pandemic brought precarity to the industry. 14 of the 32 participants (43.8%) who were employed pre‐pandemic reported decreases in income during COVID‐19, either due to work reduction or job loss. This economic impact hurt their mental health, contributing to anxiety and uncertainty.

Pat, a 22‐year‐old gay transgender man, came to the United States to live ‘free’ as a trans man. He achieved financial autonomy as a photographer pre‐pandemic. However, he lost many clients due to in‐person events being cancelled and did not know how he was going make ends meet with such a heavy reduction in income: ‘Suddenly [the events] were cut off. Nothing was supposed to be done…’ The situation was similar for Scofield, a 25‐year‐old gay man, who migrated because there were not many opportunities in his country of origin for ‘someone of my sexual orientation.’ He stated that where he was from, it could be hard for gay people to even obtain employment. In the United States, he was able to find a job working in a barber shop, where he said he saw 50–100 customers per day pre‐pandemic. However, after the onset of the pandemic, it was challenging to have even 20 customers come to the shop and some job duties were prohibited by public health protocols. He constantly worried about his boss approaching him 1 day and saying:

‘Look man, I have to lay you off for a while until the business picks up.’ That was a conversation… and I’ve heard so many people who got the same kind of conversation with their bosses…I really feared for it becoming a reality on my side.

While Pat and Scofield experienced reduced income during the pandemic, Abby, a 21‐year‐old gay man, lost his job altogether. He grew up poor, so the United States not only provided him with an opportunity to live more openly but also make more money. When he could support himself, he was able to manage, but the pandemic left him without a job and thus unable to meet his basic needs. He expressed:

I’m not in my country. I’m not near my parents … my siblings … [or] my relatives. I have no job. I don’t have my family here. I don’t have anything that could enable [me] to get this food to eat. I don’t have money to pay for my rent. It was really hard for me.

The anxiety and uncertainty related to finding and keeping employment was compounded for John, a 21‐year‐old transgender woman, who was undocumented. John, who had to hide her sexual orientation and gender identity from family and friends in her country of origin, had already been experiencing challenges since moving to the United States in 2019. She lacked documentation, which made it difficult to find a job, and was still coming to terms with her sexual orientation and gender identity. With the onset of the pandemic came additional delays in receiving the authorisations that would allow her to find a job and apply for college. As a result, she stated: ‘I don't think I have engaged in any activity that maybe brought money in.’ Even with secure immigration status, however, Eddy, a 25‐year‐old gay man with permanent residency (green card holder), still struggled because he was not yet eligible for assistance: ‘Okay, firstly, COVID has been really harsh to me, because I am an immigrant, and I have not been able to receive the government grants … I haven't been able to receive that.’

Given the financial insecurity during the pandemic, participants like Tim, a 24‐year‐old gay man, had to rely on the financial assistance of others. Tim reported that he came to the United States from his country of origin after experiencing violence and abuse and being expelled from school once he was caught kissing a boy. Coming to the United States provided him with a chance to ‘be myself,’ but because of the pandemic, Tim had to ask his father for money at a time when he thought he should be able to support himself:

Tim I used to work somewhat. I had something of my own … But when COVID‐19 came, this is when everything closed … And from then, nothing. Nothing. I cannot find other clients. And I’m now… I just am depending on my father.

Interviewer And how does it feel for you? …

T It’s not a very good feeling. Losing a job, losing some way you can get some funds and buy clothes and buy shoes, can buy electronics for your own. So the job disappears. It’s very bad, very bad feeling.

### 5.2 Theme 2: ‘When a job calls, what you have to do is answer:’ Navigating the risks of in‐person work during COVID‐19

The 17 out of 32 participants (53.1%) who continued to work outside of their homes reported extra layers of vulnerability during the pandemic. For these participants, work felt compulsory, as they had scant savings and could not rely on others' financial support. This, coupled with working service jobs, meant that these participants also had to contend with safety risks and power imbalances at work. That is, they needed to appease supervisors and customers because they could not risk losing their livelihood.

Lotus, a 23‐year‐old gay man who migrated to the United States with his family, also spoke about the need for financial autonomy and the difficult position that he was put in because of the pandemic. He had to balance his fear of getting sick (and, in turn, infecting his family) with the need to support his family of 10, which included his parents and seven siblings, some just entering pre‐school and kindergarten:

I cannot stay at home. I need to work and secure maybe a living. At the same time, we have this virus. It has been one year. I can say [it is] terrifying. But when a job calls, what you have to do is answer.

A lack of choice made participants uncomfortable about the risks they had to take to survive. Slayer, a 24‐year‐old bisexual transgender woman who worked as a child caretaker, wanted to protect herself by quarantining at home: ‘But at the same time I had that pressure of having work … and to earn a living.’ Lann, a 25‐year‐old bisexual woman, who worked as a hairstylist, described feeling scared about having constant, direct contact with many people during the pandemic, yet acknowledged that there was no other way to do her job:

Some clients don’t want to come [in] and get the [hair styling] services done. They want you to go door‐to‐door. And so that is what we have been doing, because [the clients] feel that salons and parlours are no longer as safe as they used to be … sometimes we have to do it because it is the way that you earn a living.

When at work, participants took precautions to protect themselves (e.g., social distancing, wearing masks), but they shared that their clients, who were also required to follow health and safety protocols, did not. This left them at risk of becoming ill and was indicative of how participants' socioeconomic statuses made them vulnerable to this risk. For example, Abby, who started a new job as a driver during the pandemic (and wore a mask during his interview) shared: ‘It is really hard because of the fear [of being sick].’ Despite this stress, he shared that all he could really do was: ‘say [to clients], “please to wear [your] mask … and to follow the rules strictly,” because it is our safety.’ Whether the clients abided, however, was out of his control.

Such situations exacerbated participants' fears of getting sick, but they had little recourse for protecting themselves. Lotus experienced this challenge as a plumber. Although his employer established COVID‐19 safety protocols for all employees and clients (i.e., wear masks and social distance when delivering/receiving services), Lotus encountered clients who refused to do so: ‘When you tell them they have to wear a mask, they say: ‘No, I'm in my house. Why should I wear a mask?’ In such cases, participants had to continue working under such conditions, heightening their risk of COVID‐19 transmission.

### 5.3 Theme 3: ‘It's just a very stressful time and there is no solution:’ Contending with loneliness, fear, and anxiety during COVID‐19

The pandemic's consequences for participants' mental health were far‐reaching, meaning they went beyond negative feelings associated with work and financial strain. Many reported that they were isolated because of their sexual orientation or gender identity in their countries of origin, and migrating to the United States was a way of connecting with individuals who could affirm their identities. However, COVID‐19 quarantine left participants isolated again and dealing with intense loneliness, fear, and anxiety, and sometimes this was on top of other psychological issues that they had already been struggling with.

Greg, a 24‐year‐old bisexual man, was motivated to migrate to the United States as he could not be openly bisexual in his country of origin. He hoped for ‘greener pastures … like employment, maybe having [friends], and maybe support my family back in [my country of origin].’ But this changed during the pandemic when he was stuck at home and isolated again as he was in his country of origin. Another participant, Luke, who identified as a 22‐year‐old bisexual man, also described feeling as though he was trapped in a perpetual cycle of stress and anxiety, especially as he attempted to manage the exigencies of emerging adulthood. He described that stress permeated all aspects of his life:

It’s been a lot of back and forth. Stressful back and forth. You’ve got to quarantine before, to make sure you don’t have anything before you go somewhere new. And then once you get there, you quarantine to make sure that you didn’t get anything on your way there. So it’s very stressful. But it’s also very stressful to stay in the same place. It’s just a very stressful time and there is no solution. Everything is stressful and there is nothing that you can do that isn’t stressful.

Juno, a 23‐year‐old queer non‐binary participant, migrated from a country known for being welcoming of people with diverse sexual orientations and gender identities, but grew up in a small town as a queer person of colour, which was very isolating for them. The pandemic left them feeling isolated again. These feelings were occurring at the same time that Juno was not only dealing with gender dysphoria but also their optional practical training expiring (i.e., the 1‐year period during which those with student visas are permitted to work following graduation). They expressed: ‘I think a lot of things hit on top of each other. Like my anxiety just got so much worse. I started having panic attacks.’ Having recently graduated without any full‐time employment, Juno had no health insurance and very limited access to seeing a therapist or psychiatrist. Desperate to relieve their symptoms, Juno searched online for open medical trials and signed up for an anti‐depressant trial: ‘Once I started the medication, it really did help out.’

While Juno was fortunate enough to find medication to treat their anxiety, participants such as Archie, a 25‐years‐old non‐binary pansexual individual, who also struggled with preexisting mental health issues and gender dysphoria, continued to struggle. They reported constantly ruminating about the virus, and worried that they would end up in the hospital—a place that could make their gender dysphoria worse if providers were not gender affirming:

… What if I do end up in the hospital? [It] Is a really terrifying thing, because of being hospitalised for mental illness when I was here in 2016. So … dealing with that all over again. And … I have a lot of dysphoria with regards to my body in general. So having people constantly poking and prodding at me … actually scares the living crap out of me.

Although Archie feared what would happen if they were to have to go to the hospital, John expressed anxiety about not being able to go to the hospital or seeking treatment if her mental health issues became worse or if they became physically sick. As a person without documentation, seeking treatment led to fears about what could happen if people found out she was undocumented: ‘I've been, like scared, even to seek … healthcare services, everything. I'm scared like they will discover and maybe deport me. So mostly I've been laying low.’ ‘Laying low,’ for John, meant being safe from deportation, but not necessarily being safe from the health effects of the pandemic.

### 5.4 Theme 4: ‘We would talk about how life is going…and motivate each other about daily life:’ Using online communication to manage social isolation during COVID‐19

Many pandemic‐related stressors were severe, and this intensity was unexpected, even for migrants living a new country. Despite the challenges they faced, participants found a way to be resourceful by using ICTs. For instance, to combat loneliness during the pandemic, more than half of participants (22 of the 37; 59.5%) shared that ICTs helped them stay connected to others during the pandemic. For some, like Luke, ICTs allowed them to feel less isolated:

Without social media … there is no way to engage with your friends … my best friend and roommate, he was in [his country of origin] throughout this whole semester because he couldn’t come back. The only way I could talk to him was through WhatsApp.

Viky, a 22‐year‐old transgender gay man, also used social media, Facebook in particular, to keep in touch with people. He noted: ‘I still have to socialise with my friends.’ Winy, a 24‐year‐old lesbian woman, similarly shared that she used Zoom to chat with her friends: ‘We would talk about how … life is going on, and we would motivate each other about daily life.’ These Zoom calls allowed Winy to receive moral support as she tackled the challenges of her day‐to‐day life. Slayer also relied on Zoom to maintain a sense of community with her local SGM community, which had formed an informal mutual aid group to help one another in times of need:

We are able to not just support each other mentally, we kind of support each other financially sometimes. So one of us has a challenge…then we come together and collect and look for a way [to help] … we deal with collective issues that then regard each of us.

Indeed, in the face of social isolation and pervasive uncertainty about all aspects of their lives, participants expressed that using social media could sometimes act as buffer against anxiety and depression. For example, Alan, a 24‐year‐ old gay man, started using dating apps during the pandemic:

I started using them during the COVID. Because it was very hard during the COVID to meet someone, even travelling because of the lockdown. It has been very difficult because you have no private vehicle. So I started using the technology now, like using … the dating apps … at least for me to socialise and have friends…

While the pandemic presented significant challenges to participants' ability to integrate into their local communities via in‐ person social connections, ICTs also seemed a viable method to maintain existing connections or develop new ones. Marshmellow, a 22‐year‐old bisexual woman, recalled a situation at the beginning of the pandemic. She found it difficult to meet people after having just moved to a new city. However, things changed when her new co‐workers began getting to know each other through online group chats, and ‘through that [group] chat, I made a few friends that I interact with daily.’ Another participant, Abe, a 22‐year‐old gay transgender man, also found that the Internet helped him maintain friendships during quarantine:

You don’t have to visit a friend physically, but you can just do these one at a time maybe once a month. So compared to before, this has helped me see these social media apps, like Instagram, Facebook, so you can even have a video call with your friend without even meeting personally [which] increase your time of working and reduce leisure time.

Although ICTs did help participants stay connected, buffer against anxiety and depression, and meet new people during the pandemic, there could be drawbacks. Ron, a 25‐year‐old gay man, constantly saw current event posts on Facebook and Instagram, which raised his anxiety, especially as he tried to manage his feelings about the pandemic. He finally felt he needed to avoid social media for some time: ‘I felt like it was taking a toll on me, so I kind of stopped looking at the news. It's kind of selfish, but I had to just shut it down for a little bit…’

## 6 DISCUSSION

This qualitative study examined how stress related to the COVID‐19 pandemic shaped the experiences of SGM emerging adult migrants in the United States. Participants' narratives primarily focussed on surviving at a time when much was unknown in the world; having insecure immigration status compounded this burden. These findings mirror what other SGM migrants (Harkness et al., 2021) and low‐income workers (Purkayastha et al., 2021) have experienced during the pandemic; however, the qualitative nature of this study provides additional context with which to understand the intersectional experiences of SGM emerging adult migrants. Most participants migrated to the United States to live more openly as individuals with diverse sexual orientations and gender identities. As emerging adults, many achieved financial autonomy pre‐pandemic as they explored their identities. Some were even supporting their families in their countries of origin, but pandemic‐related stressors forced them to rethink how they were going to survive. Many were scared, anxious, and uncertain, especially those who lost their jobs or significant income. Although most were employed pre‐pandemic, participants who were undocumented reported being ineligible for government assistance. A few who were permanent residents mentioned that this was the case for them, too. Indeed, evidence suggests permanent residents who had not paid taxes using a social security number or had not been in the country for 5 years were ineligible for COVID‐19 stimulus checks (Ibe, 2020). Participants struggled with the stark reality that they may have to return to their countries of origin, despite having worked hard to build lives for themselves in the United States.

Some begrudgingly received financial support from friends and family, but this came with feelings of shame. Those who considered themselves fortunate to keep their jobs felt forced to work, despite the risk of COVID‐19 infection. While participants' strength and determination helped them migrate to the United States to live more openly and make money, the pandemic left them struggling in ways that they did not expect, even as SGM emerging adult migrants. They believed they had to compromise health and safety to make a living. Additionally, if they became ill, accessing healthcare could be difficult, impossible because of their immigration status, or re‐traumatising due to previous negative experiences with providers who were not affirmative.

Findings also demonstrate the impact of pandemic restrictions on SGM emerging adult migrants' mental health. Many expressed feeling anxious, trapped, depressed, and lonely because of shelter‐in‐place and social distancing orders, suggesting that the pandemic may have compounded existing risk for poor mental health within this population. Participants were also concerned about accessing mental health services due to their immigration status. Many were ineligible, but even if free services were available, they feared that seeking services might risk detention and deportation. Transgender participants particularly expressed concerns about not being able to find affirmative providers, especially as their preexisting mental health concerns exacerbated during the pandemic. This is aligned with a previous study, which found that early in the pandemic, transgender youth were more affected by mental health challenges and had more unmet mental health and substance abuse needs than cisgender youth (Hawke et al., 2021).

Although participants contended with intense negative feelings, they demonstrated a resourcefulness that helped them manage pandemic‐related stressors. This finding is similar to studies, which have shown that SGM emerging adults exhibited resilience during the pandemic (Fish et al., 2020; Gonzalez et al., 2021), and also underscores how SGM individual's intersectional identities provide them with opportunities for resilience by connecting with others who share similar experiences (Morgan et al., 2021). Many participants noted that using ICTs was integral to deal with social isolation and loneliness. This is essential for recently arrived SGM migrants since positive close relationships can facilitate positive adjustment (Campione‐Barr et al., 2021). In this study, participants who connected with friends and tried to meet new people using ICTs shared that they felt supported, despite not being able to connect in‐person. Participants did mention a drawback to using ICTs, however. For a few, like Ron, social media constantly exposed them to news about current events, which could overwhelm them. This is consistent with a study by Harkness et al. (2022b), who found that COVID‐19 media consumption, at times, was unhelpful and stressful for Latino sexual minority men. Thus, overall findings suggest that practitioners may integrate ICTs into treatment with SGM emerging adult migrants but also work with them to recognise how ICTs can compound existing stress.

### 6.1 Limitations

The current study has limitations to consider. First, we did not recruit non‐English speaking participants. Thus, their important perspectives were not included in this study. Migrants who do not speak the official language of the destination country may experience additional challenges integrating into host societies (Nakhaie, 2020); this may have been magnified during the pandemic when access to important public health information was essential. Second, data related to how race/ethnicity shaped participants' experiences were lacking; thus, future studies should include probes to better understand intersectionality among SGM emerging adult migrants to ensure that what may be implicit becomes explicit. In some respects, the same can be said for when it came to understanding how participants' sexual and gender minority identities shaped their pandemic experiences; yet, given that participants were concerned about employment and making ends meet during the pandemic, it is not entirely surprising that their migrant identity ended up being the focus. Third, while some participants mentioned the states they resided in, we did not specifically ask participants this question to protect their privacy. However, having this information from all participants might have enriched the data by allowing us to understand how the state in which they lived also shaped their pandemic‐related experiences. Fourth, this study was cross‐sectional, and therefore, we do not know how participant experiences changed over the course of the pandemic (e.g., when restrictions were lifted and in‐person events were allowed again). Also, while vaccination rollout began in late December 2020, shortages precluded access for many until mid‐2021, especially for marginalised populations (Centers for Disease Control and Prevention, 2022). Some of the last participants to be interviewed mentioned the promise of the vaccine for restoring normalcy, but these codes did not feature prominently, likely related to the time that the majority of data were collected. Finally, while we interviewed participants until saturation, our sample did not allow for thorough examination of differences among gay men, lesbian women, and bisexual men and women. Future research should oversample these subgroups to better understand their experiences during the pandemic.

### 6.2 Implications for practice, policy, and service provision

The study has implications for practice. Findings indicate that mental health practitioners may serve a crucial role in supporting the well‐being of SGM emerging adult migrants. Alessi and Kahn (2017) outlined a framework for practice with SGM asylum seekers, which can be adapted for SGM migrants in general. This practice framework involves a trauma‐informed approach, including intersectional critical inquiry and praxis and understanding processes associated with minority stress, acculturation, and integration (Alessi & Kahn, 2017). This provides a holistic understanding of SGM migrants' experiences, especially as they attempt to manage the challenges precipitated by a public health crisis.

Our findings also have implications for service provision among SGM emerging adult migrants. While some participants could leverage their personal networks through online communication platforms for social support, fewer mentioned accessing social welfare assistance during the pandemic. One participant explicitly remarked that, due to his insecure immigration status, he was ineligible for a stimulus check. Meanwhile, many others shared that they had ‘no choice’ but to work or were unaware of what benefits were available to them. Policymakers may consider enacting and publicising a temporary entitlement programme for which migrants are eligible. This can serve as a social safety net and help control disease spread.

It has been argued that criminal legislation and immigration policies work together to disempower workers and maintain low‐cost labour (Gomberg‐Munoz, 2012). While Gomberg‐Munoz (2012) discusses the stigmatisation of Latino/a/x immigrant workers, restrictive immigration policies also perpetuate ‘historical racial and class inequalities (p. 350)’ among African migrants, who comprised 70.3% of our sample. Many migrants have few choices but to continue working low‐wage jobs in hazardous conditions, as better opportunities can be scarce. For SGM emerging adult migrants, who are just beginning to live independently, these social‐structural stressors can be compounded by stigma related to their identities (Alessi et al., 2020).

## 7 CONCLUSION

The findings of this study shed light on the stressful experiences of SGM emerging adult migrants during the COVID‐19 pandemic. Many contended with concerns about financial independence and security, which gave rise to fears about being able to cover living expenses, on top of increasing anxiety and sadness about public health protocols and social isolation. While emerging adulthood is theorised as a *gradual* transition into independence, many participants expressed that the precarity of their situations and the need to continue to work high‐risk jobs during a public health crisis were predicated on the fact that they had no one else supporting them. ICTs provided an avenue by which to connect with friends, or to access social support, but many still lacked material forms of support. It therefore remains essential that practitioners, policymakers, and researchers factor SGM emerging adult migrants' overlapping vulnerabilities into their understanding of how to better support them and to mitigate the compounding effects of public health crises on well‐being.

## CONFLICT OF INTEREST

The authors declare that there is no conflict of interest.

## Data Availability

Due to the nature of this research, supporting data are not publicly available.

## ENDNOTE

^1^
Some participants chose pseudonyms that are traditionally gendered names, which may appear inconsistent or unexpected with the gender identity that they reported. Researchers must consider how coloniality and Eurocentric frameworks have become embedded in how sex and gender are understood and how people make meaning of these constructs (Lugones, 2007; Schmidt, 2013).
